# Racial Differences in the Use of Adjuvant Chemotherapy for Breast Cancer in a Large Urban Integrated Health System

**DOI:** 10.1155/2012/453985

**Published:** 2012-05-20

**Authors:** Michael S. Simon, Lois Lamerato, Richard Krajenta, Jason C. Booza, Julie J. Ruterbusch, Sara Kunz, Kendra Schwartz

**Affiliations:** ^1^Department of Oncology, Karmanos Cancer Institute, Wayne State University, 4100 John Road, 4221 HWCRC Detroit, MI 48201, USA; ^2^Population Studies and Disparities Research Program, Karmanos Cancer Institute, Wayne State University, Detroit, MI 48201, USA; ^3^Department of Public Health Sciences, Henry Ford Health Systems, Detroit, MI 48202, USA; ^4^Department of Family Medicine and Public Health Sciences, Wayne State University, Detroit, MI 48201, USA; ^5^Wayne State University School of Medicine, Detroit, MI 48201, USA

## Abstract

*Background*. Racial differences in breast cancer survival may be in part due to variation in patterns of care. To better understand factors influencing survival disparities, we evaluated patterns of receipt of adjuvant chemotherapy among 2,234 women with invasive, nonmetastatic breast cancer treated at the Henry Ford Health System (HFHS) from 1996 through 2005. *Methods*. Sociodemographic and clinical information were obtained from linked datasets from the HFHS, Metropolitan Detroit Cancer Surveillance Systems, and U.S. Census. Comorbidity was measured using the Charlson comorbidity index (CCI), and economic deprivation was categorized using a neighborhood deprivation index. *Results*. African American (AA) women were more likely than whites to have advanced tumors with more aggressive clinical features, to have more comorbidity and to be socioeconomically deprived. While in the unadjusted model, AAs were more likely to receive chemotherapy (odds ratio (OR) 1.22, 95% confidence interval (CI) 1.02–1.46) and to have a delay in receipt of chemotherapy beyond 60 days (OR 1.68, 95% CI, 1.26–1.48), after multivariable adjustment there were no racial differences in receipt (odds ratio (OR) 1.02, 95% confidence interval (CI) 0.73–1.43), or timing of chemotherapy (OR 1.18, 95 CI, 0.8–1.74). *Conclusions*. Societal factors and not race appear to have an impact on treatment delay among African American women with early breast cancer.

## 1. Background

Despite improvements in available options for breast cancer treatment, there continues to be a considerable gap in survival between African American (AA) and white women with breast cancer [1–11]. In 2008, age-adjusted breast cancer incidence rates for white women were 129.5 per 100,000, compared to 125.6 per 100,000 for AA women, while 5-year breast cancer relative survival rates for the years 2001 through 2007 were 91.4% for white women and 77.4% for AA women http://seer.cancer.gov/csr/1975_2008/. Racial disparities in survival are even more pronounced among women diagnosed with advanced stages of disease: 5-year survival for whites versus AA of 85.2% versus 72.1% for regional, and 24.9% versus 15% for distant stage, compared to 99.3% versus 92.6% for women with local stage disease [12]. The Detroit metropolitan area has the lowest 5- and 10-year breast cancer specific survival rates compared with 10 other sites in the surveillance, epidemiology, and end results program (SEER) [7], as well as one of the largest AA populations, making Detroit an ideal area in which to study factors influencing racial differences in breast cancer survival.

Adjuvant chemotherapy and hormonal therapy have had a dramatic impact on breast cancer survival, and in order to optimize longevity, it is critical for patients to receive treatment according to standard clinical guidelines [13, 14]. Previous reports on patterns of care have indicated that AA women are less likely to receive standard breast cancer treatment compared with white women [2, 5, 6, 8, 15–17]. Disparities in receipt of treatment have been shown to be associated with both lack of referral to specialists, and/or other barriers including financial and sociodemographic issues [4, 18]. In regards to adjuvant chemotherapy, studies have shown that comorbidity and low socioeconomic status both negatively influence receipt of adjuvant chemotherapy [7, 19] and others have highlighted the impact of treatment delay [8, 20, 21] or receipt of nonstandard chemotherapy regimens [15, 19, 20] as factors associated with inadequate care.

 We hypothesized that racial disparities in breast cancer survival may be at least in part due to differences in the receipt of standard adjuvant chemotherapy as defined by national treatment guidelines. In order to address this question, we evaluated patterns of breast cancer care provided at the Henry Ford Health System (HFHS), a large integrated health system serving southeastern MI. The goal of this study was to assess patterns of adjuvant chemotherapy administration among women with invasive, nonmetastatic breast cancer comparing AA and white women, and focusing on receipt of standard chemotherapy, duration of treatment, and timing of treatment in relationship to diagnosis.

## 2. Methods

### 2.1. Study Design

 This study consisted of a descriptive analysis of adjuvant chemotherapy received by AA and white women diagnosed with invasive, nonmetastatic breast cancer at the HFHS between January 1, 1996 and December 31, 2005. HFHS is a large urban integrated health system located in southeast Michigan founded in 1915 to provide for the health care needs of the city of Detroit and surrounding metropolitan area. HFHS currently consists of 5 hospitals, anchored by Henry Ford Hospital, a 903 bed tertiary care, research and teaching facility; and 36 ambulatory care facilities including 5 sites located within the city of Detroit, and 31 sites located in Wayne (outside of Detroit), Macomb, Oakland, and Washtenaw counties. A single lifetime medical record number (MRN) is used throughout the system to provide continuity of record keeping and medical care. For the purposes of this project, patient sociodemographic, clinical, and treatment information was derived through analyses of linked datasets using the HFHS administrative databases, the Metropolitan Detroit Cancer Surveillance System (MDCSS), and the U.S. Census Bureau. The MDCSS is home for the Detroit SEER registry, which registers all cancers of residents from Wayne, Oakland, and Macomb counties.

 In the current study, case records were matched from the SEER and HFHS databases using MRN, social security number (SSN), last name, and date of birth. Records that matched for only one variable were manually reviewed to look for character or punctuation errors in other nonmatched fields. Matching resulted in 3,630 record matches. We excluded matches with unknown American Joint Cancer Committee (AJCC) stage (*n* = 51); first breast surgery at another institution (*n* = 147); history of a prior malignancy within 6 months of breast cancer diagnosis (*n* = 52); duplicate records (*n* = 3); histology code indicating non-breast origin (*n* = 1); stage IV disease (*n* = 978), other race (*n* = 45); no definitive breast surgery (*n* = 52); and receipt of neoadjuvant chemotherapy (*n* = 67). These exclusions resulted in a study population of 2,234 (61.5%) white and AA women treated for invasive, nonmetastatic breast cancer at the HFHS.

### 2.2. Measurement of Variables

 Detailed information on breast cancer treatment, clinical, and socio-demographic data were derived from the HFHS and SEER database and information on neighborhood-level economic deprivation (see deprivation index below) was obtained form the U.S. Census Bureau. All primary breast surgery consisting of lumpectomy (partial mastectomy) or mastectomy (modified radical mastectomy, radical mastectomy, or simple mastectomy) and standard axillary lymph node dissection was performed at the HFHS. Guidelines from the National Comprehensive Cancer Network (NCCN) corresponding to the years of diagnosis were used to define standard adjuvant chemotherapy treatment recommendations according to AJCC stage [22–24]. Data on type of chemotherapy, number of chemotherapy cycles, and dates of diagnosis and chemotherapy administration were used to assess whether or not each patient received standard NCCN recommended adjuvant chemotherapy and the timing of treatment.

 Patient and clinical characteristics included race (from the medical record listing), age at diagnosis, tumor size, lymph node positivity, histology, grade, and estrogen and progesterone receptor (ER and PR) status. Insurance status was available from the HFHS records and was classified based on the most frequent insurance charged for each treatment visit, and categorized into 3 groups (private, Medicare, and other, including uninsured). Comorbidity was assessed using the Charlson comorbidity index (CCI) a prospectively verified method for classifying comorbid medical conditions which could affect the risk of mortality in longitudinal studies [25, 26]. CCI was calculated using all medical diagnoses for 1 year prior, through one-month post-breast cancer diagnosis. Economic deprivation was measured through the use of a composite measure at the census tract level using a modification of the material deprivation index of Klassen et al., which is more robust than poverty measures [27]. The material deprivation index captures multiple dimensions of the economic and social conditions of neighborhoods, as well as the social isolation of the residents. The variables included in the index are defined as (1) the proportion of households with no vehicle available; (2) proportion of households with no telephone available; (3) proportion of the population 16 years of age and older that is unemployed; (4) proportion of the population living in a crowded residence (more than 1 person per room); and (5) proportion of the population living below the poverty level. The data are contained in the 2000 US Census Bureau Summary File 3, Summary Level 140, Tables H44, H43, P43, H20, and P87, respectively, [http://www.ncbi.nlm.nih.gov/pubmed/15313082/]. The index is calculated by adding the value of each variable and dividing by five in order to produce a single index value, range 0 to 1, with 0 representing no economic deprivation and 1 absolute deprivation. For the purpose of our analysis, DI was categorized into quintiles, based on the distribution of deprivation in the tricounty area (Q1 < 0.022, 0.022 ≤ Q2 < 0.035, 0.035 ≤ Q3 < 0.056, 0.056 ≤ Q4 < 0.142, 0.142 ≤ Q5 < 0.531).

### 2.3. Statistical Analysis

The clinical and sociodemographic characteristics of AA and white women with invasive, nonmetastatic breast cancer were compared by chi-square tests for categorical variables and Student's *t*-tests for continuous variables. Separate analyses were conducted to determine racial differences in the use of standard chemotherapy (yes versus no), timing of chemotherapy as determined by the date of diagnosis and the date of chemotherapy initiation (dichotomized using the sample median, 60 days) for cases where detailed chemotherapy records were available and completion of standard chemotherapy (i.e., completing the NCCN recommended number of cycles of treatment).

Odds ratios (ORs) for receipt of chemotherapy for AA versus white women and 95% confidence intervals (CIs) were estimated using unconditional logistic regression analyses. Race, age at diagnosis, tumor size, lymph node positivity, hormone receptor status, tumor grade, CCI, deprivation index, and insurance status were assessed individually and in multivariable adjusted models. Unconditional logistic regression was also used to estimate the odds of beginning chemotherapy within 60 days of the date of diagnosis. The analyses consisted of three models, first adjusting for clinical factors only (race, age, tumor size, lymph node positivity, hormone receptor status, tumor grade, and CCI), second adjusting for societal factors (race, deprivation index, and insurance status), and third adjusting for all listed variables. The purpose of performing three different models was to determine whether clinical versus societal factors had a greater impact on racial differences in receipt of adjuvant chemotherapy or in timing of chemotherapy. All regression models were run with and without a clustering correction for census tract.

## 3. Results

Table 1 shows the clinical and socio-demographic characteristics of women with invasive, non-metastatic breast cancer in the HFHS study cohort categorized by race. The average age at diagnosis was 61.2 years (SD 14 years), and the majority of women were diagnosed with early stage breast cancer (52.0% were AJCC Stage I and 40.8% were AJCC Stage II) and 71% were axillary lymph node negative. Most of the breast tumors were ductal (77%), well or moderately differentiated (60%), and ER and/or PR positive (74%), and roughly 2/3 of the women had no other listed medical conditions as defined by the CCI. The majority of patients in the study cohort had lumpectomy or partial mastectomy (66%), 44% received adjuvant chemotherapy and 51% of the women had private insurance, followed by Medicare (35%) and other (4%).

Table 2 shows the proportion of persons or the percent of households in each quintile of deprivation index by the parameters used to define the variable. Individuals living in the highest quintile (indicating the most deprivation), quintile 5 (Q5), had 22.5 percent unemployment, compared with 2.5% in quintile 1. Similarly, for quintile 5, 35% of the population lived under the poverty level compared with 2.1% in quintile 1. For households in quintile 5, almost 30% had no vehicle, 10% had no telephone, and 10% were defined as overcrowded. About 40% of the study population lived in a neighborhood defined by deprivation index quintiles 4 and 5 indicating that a sizable portion of our study population lived in the most socioeconomically deprived neighborhoods.

There were no significant racial differences in age at diagnosis (Table 1), however, AA women were significantly more likely than white women to have tumors that were larger size (40 versus 31% ≥ 2.0 cm; *P* < 0.001), lymph node positive (34 versus 27%; *P* < 0.001), ER/PR negative (29 versus 19%; *P* < 0.001), and poorly differentiated or undifferentiated (44 versus 32%; *P* < 0.001). AA women were more likely compared to white women to have had a mastectomy (37 versus 32%; *P* = 0.009) and to have received adjuvant chemotherapy (48 versus 43%; *P* = 0.028). AA women were also more likely to have a higher CCI score, to live in an area with a higher deprivation index (45% versus 5% resided in quintile 5; *P* ≤ 0.001), and were less likely to have private insurance (58 versus 63%: *P* = 0.020).

Table 3 shows the results of the logistic regression analysis for predictors of receipt of adjuvant chemotherapy. The unadjusted model for race revealed that AA women were significantly more likely to receive adjuvant chemotherapy than whites (odds ratio (OR) 1.22, 95% confidence interval (CI) 1.02–1.46). Other significant predictors of receipt of chemotherapy included tumor size, lymph node positivity, and ER/PR negativity. Older women and women with Medicare or a higher CCI were less likely to receive chemotherapy. In the model adjusting for all listed predictor variables, race was no longer a significant predictor for receipt of adjuvant chemotherapy (OR 1.02, 95% CI, 0.73–1.43). In an attempt to better understand the role of selected clinical versus societal factors on the effect of race on receipt of adjuvant chemotherapy, we analyzed two other models, first adjusting for clinical factors alone, and secondly adjusting for societal factors. In the model adjusting for clinical factors, race was no longer a significant predictor for the receipt of adjuvant chemotherapy (OR 0.90, 95% CI, 0.69–1.17), however, in the model adjusting for only societal factors, the odds of receipt of chemotherapy for AA versus white women was larger (OR 1.60, 95% CI, 1.26–2.04) than was seen in the unadjusted model.

Table 4 shows the results of racial differences in the use of NCCN standard adjuvant chemotherapy regimens and timing of chemotherapy administration. There were no racial differences in whether an NCCN standard adjuvant chemotherapy regimen was administered, or in the completion of the recommended number of chemotherapy cycles. There was, however, a delay in the initiation of adjuvant chemotherapy for AA women compared with white women. The average time from diagnosis to initiation of chemotherapy for white women was 67.9 days (S.D. 38.6) compared to 73.2 (S.D. 36.4) for AA women, *P* = 0.049. When time to adjuvant chemotherapy was stratified at 60 days (the sample median), white women were more likely to be treated prior to 60 days (55%) compared to AA women (43%), *P* < 0.001.

Table 5 shows the results of the logistic regression model evaluating factors that predict timing of adjuvant chemotherapy administration. Unadjusted analyses showed that AA women were more likely than white women to have a delay in receipt of chemotherapy beyond 60 days (OR 1.68, 95% CI, 1.26–2.23), while in the fully adjusted model, there were no significant racial differences in time to chemotherapy initiation (OR 1.18, 95% CI, 0.80–1.74). Again to better understand the role of selected clinical versus societal factors on the effect of race on timing of adjuvant chemotherapy, we analyzed a “clinical model” followed by a “societal model.” While in the clinical model, AA women were still more likely to have a delay in receipt of adjuvant chemotherapy compared to white women (OR 1.77, 95% CI, 1.32–2.38), in the model adjusted for societal factors, race no longer had a significant impact on receipt of adjuvant chemotherapy (OR 1.15, 95% CI, 0.79–1.67).

## 4. Discussion

 While breast cancer survival rates continue to improve over time http://seer.cancer.gov/csr/1975_2008/, there remains a marked discrepancy in survival by race, [1, 3, 4, 11, 12] with even greater differences in survival seen for AA and white women diagnosed with advanced disease [12]. The availability of new adjuvant chemotherapy and hormonal therapy regimens have had an important impact on the improvement in survival over time [13, 14], however, access to high-quality oncologic care is a key determinant of whether women receive the recommended standard treatment. It is likely that racial disparities in the receipt or timing of adjuvant chemotherapy could have a negative impact on breast cancer survival.

 In this report, we present data on patterns of adjuvant chemotherapy administration for invasive, nonmetastatic breast cancer from a large urban health care system where presumably all individuals who are part of that system have equal access to the same quality medical care. In fact, in the unadjusted analysis, AA women were more likely to receive adjuvant chemotherapy then their white counterparts, however, this difference disappeared in the multivariable adjusted model and was largely accounted for by the fact that AA women were more likely to present with larger and more aggressive tumors at diagnosis. Of particular interest, our unadjusted results demonstrated a delay in initiation of chemotherapy for AA compared with white women, however, this difference was also accounted for in the multivariable adjusted model, and largely accounted for by societal factors. In fact, in the model adjusting for societal variables, deprivation index and insurance status offset the effect of race on timing of receipt. In the model adjusting for clinical factors, only mastectomy was associated with chemotherapy delay; however, adjusting for it did not affect racial differences in timing. It is understandable that mastectomy may delay start of chemotherapy, regardless of race as there may be a longer recovery period compared to breast conserving surgery. The relationship between societal factors and delay in receipt of chemotherapy suggest that while a high-quality medical system is necessary for the provision of medical care, other related factors such as poverty and social mobility, can have an important impact on whether individuals are able to access and benefit from the medical care system.

 Racial disparities in the receipt of breast cancer treatment has been well documented in the literature including studies revealing disparities in receipt of adjuvant hormonal and chemotherapy [28], diagnostic and treatment delays [8, 21], inadequate dosing [7, 8, 15], and receipt of nonstandard treatment [2]. Others have reported on factors other than race which impact receipt of treatment and have demonstrated the relationship between socioeconomic status and type of treatment [28] and chemotherapy dose [19] and the influence of socioeconomic status on breast-cancer-related mortality [4, 7, 18]. Importantly, the higher prevalence of comorbid medical conditions seen in AA women has a large influence on breast cancer mortality [10, 11]. Our results are consistent with the literature suggesting that influences other than race affect timing of adjuvant chemotherapy, and highlight the role of poverty and deprivation on timely receipt of recommended treatment.

 Strengths of this study include the inclusion of women enrolled in a large integrated urban heath care system which provides uniform access to high-quality medical care. In addition, the linked HFHS and SEER database allowed for availability of detailed and accurate clinical, demographic, and treatment data including details on adjuvant chemotherapy received. Our measure of socioeconomic deprivation was a sophisticated measure developed through the linkage with U.S. Census data, however, the derived deprivation index was not based on factors specific to the individual patient such as income, education, or family support, and may therefore be subject to misclassification.

In conclusion, race had no direct impact on receipt of adjuvant chemotherapy or timing of chemotherapy among a cohort of women treated at a large urban integrated health care system in Detroit. The fact that AA women were more likely to receive adjuvant chemotherapy in the unadjusted model was largely explained by the more advanced stage at diagnosis among AAs that suggests the need for better screening and access to early treatment interventions. Delay in receipt of chemotherapy among AA women was largely explained by societal factors which likely have a direct effect on access to care. However, the delay was on average less than one week and may not have had significant clinical impact. Nevertheless, it serves to remind health care providers of the importance of making health care accessible to all.

## Figures and Tables

**Table 1:** Distribution of clinical and sociodemographic features of the HFHS study cohort stratified by race.

	Both races *N *(%)	White *N *(%)	African American *N *(%)	*P* value*
Total	2,234	1,499	735	
Age at diagnosis				0.260
<50	526 (23.5%)	346 (23.1%)	180 (24.5%)	
50–64	723 (32.4%)	474 (31.6%)	249 (33.9%)	
65+	985 (44.1%)	679 (45.3%)	306 (41.6%)	
Mean (std)	61.2 (14.0)	61.6 (13.9)	60.5 (14.1)	0.091
Tumor size				<0.001
<2 cm	1449 (64.9%)	1010 (67.4%)	439 (59.7%)	
2.1–5 cm	648 (29.0%)	407 (27.2%)	241 (32.8%)	
>5 cm	102 (4.6%)	54 (3.6%)	48 (6.5%)	
Unknown	35 (1.6%)	28 (1.9%)	7 (1.0%)	
Lymph node status				<0.001
Negative	1575 (70.5%)	1091 (72.8%)	484 (65.9%)	
Positive	659 (29.5%)	408 (27.2%)	251 (34.1%)	
AJCC stage				0.001
I	1162 (52.0%)	823 (54.9%)	339 (46.1%)	
II	911 (40.8%)	575 (38.4%)	336 (45.7%)	
III	161 (7.2%)	101 (6.7%)	60 (8.2%)	
ER/PR receptors				<0.001
ER+/PR+	1404 (62.8%)	999 (66.6%)	405 (55.1%)	
ER+/PR−	200 (9.0%)	125 (8.3%)	75 (10.2%)	
ER−/PR+	41 (1.8%)	29 (1.9%)	12 (1.6%)	
ER−/PR−	496 (22.2%)	280 (18.7%)	216 (29.4%)	
Unknown	93 (4.2%)	66 (4.4%)	27 (3.7%)	
Histology				0.052
Ductal	1730 (77.4%)	1141 (76.1%)	589 (80.1%)	
Lobular	197 (8.8%)	146 (9.7%)	51 (6.9%)	
Mixed	134 (6.0%)	98 (6.5%)	36 (4.9%)	
Other	173 (7.7%)	114 (7.6%)	59 (8.0%)	
Grade				<0.001
Well differentiated	414 (18.5%)	307 (20.5%)	107 (14.6%)	
Moderate/Poor/ Undifferentiated	1725 (77.2%)	1125 (75.1%)	600 (81.6%)	
Unknown	95 (4.3%)	67 (4.5%)	28 (3.8%)	
Surgery				0.009
Lumpectomy/ Partial Mastectomy	1481 (66.3%)	1021 (68.1%)	460 (62.6%)	
Mastectomy	753 (33.7%)	478 (31.9%)	275 (37.4%)	
Adjuvant chemotherapy				0.028
No	1247 (55.8%)	861 (57.4%)	386 (52.5%)	
Yes	987 (44.2%)	638 (42.6%)	349 (47.5%)	
Charlson comorbidity index (CCI)^a^				<0.001
None	1459 (65.3%)	1019 (68.0%)	440 (59.9%)	
1	440 (19.7%)	275 (18.3%)	165 (22.4%)	
2	136 (6.1%)	82 (5.5%)	54 (7.3%)	
3+	111 (5.0%)	52 (3.5%)	59 (8.0%)	
Unknown	88 (3.9%)	71 (4.7%)	17 (2.3%)	
Deprivation index^b^				<0.001
Q1	420 (18.8%)	406 (27.1%)	14 (1.9%)	
Q2	436 (19.5%)	395 (26.4%)	41 (5.6%)	
Q3	478 (21.4%)	417 (27.8%)	61 (8.3%)	
Q4	501 (22.4%)	211 (14.1%)	290 (39.5%)	
Q5	397 (17.8%)	68 (4.5%)	329 (44.8%)	
Unknown	2 (0.1%)	2 (0.1%)	0 (0.0%)	
Insurance				0.020
Private	1370 (61.3%)	947 (63.2%)	423 (57.6%)	
Medicare	780 (34.9%)	503 (33.6%)	277 (37.7%)	
Other^c^	84 (3.8%)	49 (3.3%)	35 (4.8%)	

*P-value calculations do not include unknown values.

^a^The Charlson comorbidity index (CCI) is a prospectively verified method for classifying comorbid medical conditions which could affect the risk of mortality in longitudinal studies.

^b^Neighborhood economic deprivation was assessed through a material deprivation index (DI) that captures multiple dimensions of the economic and social conditions of neighborhoods including unemployment, poverty, residential overcrowding, as well as telephone and automobile availability. Quintile 1 indicates less economic deprivation.

^c^The other category included 23 self-pay or uninsured, 24 government sponsored MHO, 29 Medicaid, 2 CHAMPUS and 2 insurance pending.

**Table 2:** Proportion of persons or households in each quintile of deprivation index^a^ by the five parameters used to create the deprivation index.

DI Quintile	Unemployment^b^	No vehicle^c^	Poverty^b^	No Telephone^c^	Overcrowding^c^
Q5	22.5	29.9	35.1	10.4	9.5
Q4	9.2	12.7	14.6	3.4	5.4
Q3	4.8	6.7	6.3	1.5	2.9
Q2	3.7	4.1	3.9	0.7	1.8
Q1	2.5	1.9	2.1	0.4	0.9

^a^The deprivation index (DI) is a measure of socioeconomic status that captures multiple dimensions of the economic and social conditions of neighborhoods including unemployment, poverty, overcrowding, telephone, and automobile availability. The DI can range from 0 to 1, with a value of 0 indicating no deprivation (i.e., no unemployment, all households have a phone and automobile, no individual lives below poverty, and the presence of households with more than one room per person, and a value of 1 indicating maximum deprivation.

^b^% of persons.

^c^% of households.

**Table 3:** Logistic regression analysis of predictors of the receipt of adjuvant chemotherapy.

	Unadjusted OR (95% C.I.)	Clinical factors OR* (95% C.I.)	Societal factors OR* (95% C.I.)	Adjusted OR* (95% C.I.)
African American	1.22 (1.02–1.46)	0.88 (0.67–1.14)	1.60 (1.26–2.04)	1.01 (0.72–1.42)
Age at diagnosis^a^	0.92 (0.91–0.93)	0.91 (0.90–0.92)		0.92 (0.91–0.94)
Large tumor^b^	4.66 (3.85–5.63)	3.71 (2.81–4.89)		3.76 (2.84–4.98)
Lymph node positive	8.99 (7.24–11.16)	10.18 (7.54–13.74)		10.28 (7.58–13.94)
ER−/PR−	3.68 (2.97–4.56)	3.49 (2.57–4.74)		3.56 (2.61–4.85)
Moderate/High grade	3.37 (2.63–4.32)	1.40 (1.00–1.98)		1.44 (1.02–2.04)
CCI^c^	0.68 (0.61–0.76)	0.93 (0.79–1.10)		0.95 (0.81–1.13)
Deprivation index^d^	0.95 (0.89–1.01)		0.91 (0.84–0.99)	0.95 (0.85–1.07)
Medicare^e^	0.18 (0.15–0.22)		0.18 (0.15–0.22)	0.48 (0.34–0.68)
Other insurance^e^	0.66 (0.43–1.03)		0.68 (0.44–1.07)	0.43 (0.22–0.85)

*Models adjusted for all listed variables.

^a^Continuous variable.

^b^≤2 cm is the referent.

^c^Charlson comorbidity index; continuous variable capped at 3.

^d^Quintiles, the least deprived area (quintile 1) is the referent.

^e^Private insurance is the referent.

**Table 4:** Racial differences in the use of standard adjuvant chemotherapy and timing of chemotherapy administration.

	Total		White	African American	*P* value
Standard regimen^a^					
No	210 (26.2%)		128 (25.9%)	82 (26.6%)	
Yes	593 (73.8%)		367 (74.1%)	226 (73.4%)	0.811
Time to chemotherapy^b^					
0–60 days	405 (50.4%)		274 (55.4%)	131 (42.5%)	
>60 days	398 (49.6%)		221 (44.7%)	177 (57.5%)	<0.001
Completed the recommended number of cycles or more^a,c^					
No	172 (29.0%)		106 (28.9%)	66 (29.2%)	
Yes	421 (71.0%)		261 (71.1%)	160 (70.8%)	0.933

^a^Standard regimen as defined by the NCCN guidelines as of the date of breast cancer diagnosis.

^b^Time to chemotherapy based on the time period from date of diagnosis to the date of initiation of adjuvant chemotherapy.

^c^Calculated only for women who received a standard regimen.

**Table 5:** Logistic regression analysis of predictors of the timing of adjuvant chemotherapy.

	Unadjusted OR (95% C.I.)	Clinical Factors OR* (95% C.I.)	Societal Factors OR* (95% C.I.)	Adjusted OR* (95% C.I.)
African American	1.68 (1.26–2.23)	1.77 (1.31–2.38)	1.15 (0.79–1.67)	1.18 (0.80–1.74)
Age at diagnosis^a^	1.01 (1.00–1.03)	1.01 (1.00–1.03)		1.01 (0.99–1.02)
Comorbidity index^b^	0.97 (0.79–1.20)	0.87 (0.70–1.08)		0.86 (0.68–1.07)
Mastectomy^c^	1.45 (1.09–1.92)	1.42 (1.06–1.91)		1.38 (1.02–1.85)
Deprivation index^d^	1.27 (1.15–1.40)		1.22 (1.07–1.39)	1.23 (1.08–1.41)
Medicare^e^	1.73 (1.14–2.61)		1.58 (1.04–2.40)	1.34 (0.81–2.23)
Other insurance^e^	1.38 (0.64–3.00)		1.11 (0.50–2.43)	1.08 (0.49–2.40)

*Models adjusted for all listed variables.

^a^Continuous variable.

^b^Charlson comorbidity index; continuous variable capped at 3.

^c^The reference group is lumpectomy or partial mastectomy.

^d^Quintiles, the least deprived area (quintile 1) is the referent.

^e^Private insurance is the referent.

## References

[1] Clegg LX, Li FP, Hankey BF, Chu K, Edwards BK (2002). Cancer survival among US whites and minorities: a SEER (Surveillance, Epidemiology, and End Results) Program population-based study. *Archives of Internal Medicine*.

[2] Li CI, Malone KE, Daling JR (2003). Differences in breast cancer stage, treatment, and survival by race and ethnicity. *Archives of Internal Medicine*.

[3] Ghafoor A, Jemal A, Ward E, Cokkinides V, Smith R, Thun M (2003). Trends in breast cancer by race and ethnicity. *Ca-A Cancer Journal for Clinicians*.

[4] Ward E, Jemal A, Cokkinides V (2004). Cancer disparities by race/ethnicity and socioeconomic status. *Ca-A Cancer Journal for Clinicians*.

[5] Simon MS, Banerjee M, Crossley-May H, Vigneau FD, Noone AM, Schwartz K (2006). Racial differences in breast cancer survival in the Detroit Metropolitan area. *Breast Cancer Research and Treatment*.

[6] Hershman D, McBride R, Jacobson JS (2005). Racial disparities in treatment and survival among women with early-stage breast cancer. *Journal of Clinical Oncology*.

[7] Grann V, Troxel AB, Zojwalla N, Hershman D, Glied SA, Jacobson JS (2006). Regional and racial disparities in breast cancer-specific mortality. *Social Science and Medicine*.

[8] Hershman DL, Unger JM, Barlow WE (2009). Treatment quality and outcomes of African American versus white breast cancer patients: retrospective analysis of southwest oncology studies S8814/S8897. *Journal of Clinical Oncology*.

[9] Lund MJ, Trivers KF, Porter PL (2009). Race and triple negative threats to breast cancer survival: a population-based study in Atlanta, GA. *Breast Cancer Research and Treatment*.

[10] Braithwaite D, Tammemagi CM, Moore DH (2009). Hypertension is an independent predictor of survival disparity between African-American and white breast cancer patients. *International Journal of Cancer*.

[11] Tammemagi CM, Nerenz D, Neslund-Dudas C, Feldkamp C, Nathanson D (2005). Comorbidity and survival disparities among black and white patients with breast cancer. *Journal of the American Medical Association*.

[12] Howlader N, Noone AM, Krapcho M (2011). *SEER Cancer Statistics Review 1975–2008*.

[13] Glück S, Mamounas T (2010). Improving outcomes in early-stage breast cancer. *Oncology (Williston Park, N.Y.)*.

[14] Carlson RW (2003). NCCN breast cancer clinical practice guidelines in oncology: an update. *JNCCN: Journal of the National Comprehensive Cancer Network*.

[15] Griggs JJ, Sorbero MES, Stark AT, Heininger SE, Dick AW (2003). Racial disparity in the dose and dose intensity of breast cancer adjuvant chemotherapy. *Breast Cancer Research and Treatment*.

[16] Du W, Simon MS (2005). Racial disparities in treatment and survival of women with stage I-III breast cancer at a large academic medical center in metropolitan Detroit. *Breast Cancer Research and Treatment*.

[17] Bradley CJ, Given CW, Roberts C (2002). Race, socioeconomic status, and breast cancer treatment and survival. *Journal of the National Cancer Institute*.

[18] Byers TE, Wolf HJ, Bauer KR (2008). The impact of socioeconomic status on survival after cancer in the United States: findings from the National Program of Cancer Registries patterns of care study. *Cancer*.

[19] Griggs JJ, Culakova E, Sorbero MES (2007). Social and racial differences in selection of breast cancer adjuvant chemotherapy regimens. *Journal of Clinical Oncology*.

[20] Hershman D, Weinberg M, Rosner Z (2003). Ethnic neutropenia and treatment delay in African American women undergoing chemotherapy for early-stage breast cancer. *Journal of the National Cancer Institute*.

[21] Gorin SS, Heck JE, Cheng B, Smith SJ (2006). Delays in breast cancer diagnosis and treatment by racial/ethnic group. *Archives of Internal Medicine*.

[22] National Comprehensive Cancer Network (NCCN) (2003). *NCCN Clinical Practice Guidelines in Oncology: Breast Cancer, Version 1*.

[23] National Comprehensive Cancer Network (NCCN) (2000). *NCCN Clinical Practice Guidelines in Oncology: Breast Cancer, Version 1*.

[24] Carlson RW, Goldstein LJ, Gradishar WJ (1996). NCCN Breast Cancer Practice Guidelines. The National Comprehensive Cancer Network. *Oncology*.

[25] Charlson ME, Pompei P, Ales KL, MacKenzie CR (1987). A new method of classifying prognostic comorbidity in longitudinal studies: development and validation. *Journal of Chronic Diseases*.

[26] Deyo RA, Cherkin DC, Ciol MA (1992). Adapting a clinical comorbidity index for use with ICD-9-CM administrative databases. *Journal of Clinical Epidemiology*.

[27] Klassen AC, Curriero FC, Hong JH (2004). The role of area-level influences on prostate cancer grade and stage at diagnosis. *Preventive Medicine*.

[28] Banerjee M, George J, Yee C, Hryniuk W, Schwartz K (2007). Disentangling the effects of race on breast cancer treatment. *Cancer*.

